# Multisectoral contributions to health security and formal policy availability at the community level in Nigeria

**DOI:** 10.3389/fpubh.2025.1505383

**Published:** 2025-02-28

**Authors:** Enyi Etiaba, Prince Agwu, Lesong Conteh, Obinna Onwujekwe

**Affiliations:** ^1^Health Policy Research Group, Department of Pharmacology and Therapeutics, College of Medicine, University of Nigeria, Enugu, Nigeria; ^2^Department of Health Administration and Management, College of Medicine, University of Nigeria, Enugu, Nigeria; ^3^Department of Social Work, University of Nigeria, Enugu, Nigeria; ^4^School of Humanities, Social Sciences, and Law, University of Dundee, Dundee, United Kingdom; ^5^Department of Health Policy, London School of Economics and Political Science, London, United Kingdom

**Keywords:** multisectoral collaboration, community level, health security, policy, social determinants of health

## Abstract

**Introduction:**

Multisectoral plans and actions at the community level are one of the strategies that are deployed in the primary healthcare (PHC) system for improving the health and wellbeing of the people and also a means of addressing the social determinants of health. Multisectoral actions are also a means of implementing the Health in All Policies (HiAP) policy directions, which Nigeria has agreed to implement. However, there is a paucity of knowledge on the level of multisectoral involvement to ensure health security and promotion at the community level. This paper provides new knowledge on what multisectoral activities for health are undertaken at the community level and what can be done to strengthen them towards achieving universal health coverage in Nigeria. It elaborates on previous and current levels of multisectoral collaboration (MSC) activities for health at the community level.

**Methods:**

A qualitative cross-sectional case study of three contextually different states in northern (Kano) and southern (Akwa Ibom and Anambra) states in Nigeria. Conceptually, the study was guided by the Expanded Health Systems framework, which recognises potential combinations of collaborations between the non-health sector and other societal partnerships (CSOs, NGOs, community groups, and informal health providers) to directly contribute to community health or indirectly through one or more social determinants of health. The study was also guided by the WHO PHC operational framework, which proposes multisectoral action as one of three key approaches to UHC. Data were collected and triangulated through 103 in-depth interviews with policymakers (health and non-health sectors), formal and informal health providers, and community leaders; 12 focus group discussions with community members (service users) and a review of health and non-health sector policy documents. Thematic data analysis was undertaken.

**Results:**

Several community and household-level activities were identified as having been borne out of multisectoral actions. Most activities were initiated by health sector stakeholders in health, whereas others were initiated by non-health sectors (education, environment, agriculture, security, women affairs, social welfare, nutrition, water, sanitation, and hygiene—WASH) or communities. The multisectoral activities contributed to primary healthcare activities and the health security of communities, directly or indirectly, through improving one or several social determinants of health (water supply, housing, environment, security, food, and nutrition). However, most activities, which involved collaborative engagements with non-health sectors, were not backed by any formal, explicit non-health sectoral policies or guidelines. Rather, they were organically initiated and developed to support health security. The support of community leaders and groups facilitated the initiation and sustenance of multisectoral activities, whilst inadequate formal policy backing and funding were the major constraints. Although there are calls in the country for non-health sectors to mainstream health in their sectors, there is yet no clearly established framework or guidelines through which this can be implemented and sustained. A multisectoral action plan for non-communicable diseases has been developed but has not been implemented and evaluated.

**Conclusion:**

Multisectoral collaboration for health at the community level is important for harnessing resources from outside the health sector that will be used to enhance the health security of communities. Such MSC is potentially a powerful tool for strengthening primary healthcare, towards UHC, and achieving SDG3, as shown by our findings. However, entrenched and sustained MSC should be undertaken through explicitly intentional policy reforms and their implementation through identifying, promoting, and financing MSC actions.

## Introduction

A key aspired innovative approach in the last decades has been multisectoral action for health, captured as Health in All Policies (1). Multisectoral actions for health are those actions taken by non-health sectors to protect the health of the population (2). A study of ten African countries by the World Health Organization (WHO), focussing on the capacities of communities to contribute to, and engage in health service delivery, recommended the participation of other sectors and the community, in addition to the health sector, to achieve “*an acceptable level of health”* (3).

Multisectoral policy and action is one of the component strategies of primary healthcare for achieving health and wellbeing and specifically addresses the determinants of, and threats to, health (4). This is in acknowledgement that many of the social, economic, geographic, and environmental determinants of health are beyond the policy boundaries of the health sector. Hence, various global and regional calls have been made to address these factors through the Health in All Policies (HiAP) approach (1–5).

Failure to consider multiple factors, social, economic, political, environmental, and social determinants beyond the health sector, is argued to have contributed to many developing countries not achieving the Millennium Development Goals (MDGs), especially the maternal and child health-related goals (6), and in general, noted that sectoral action beyond the health sector is indispensable in addressing the structural determinants of health (7).

Multisectoral action for health has been noted to be ‘atheoretical’ (8) and frameworks predominantly disease-specific (9–11). However, the Committee on Social Determinants of Health framework recognises individuals (households) and communities as the micro- and meso-level entry points, respectively, for multisectoral action in addressing SDH (7).

Community health has been referred to as the health status of this defined group of people and the actions and conditions, both private and public (governmental), to promote, protect, and preserve their health and wellbeing (12)-P 6]. Whilst formal healthcare, mainly curative, is provided at health facilities, many services, especially preventive and promotive health services, are also provided in the communities, from the household level to health outreaches (13). Whether these occur in the form of a community health system (CHS) outside of formal structures (14, 15), or as one of the modalities of a broader formal primary care unit (16), it has been argued that “Africa’s key strength lies in the communities whose potential should be unlocked to build cost effective and sustainable bottom-up health systems founded on Primary Health Care (PHC)...”[P2] (17). Both definitions also recognise the crucial role of multiple stakeholders, within and outside the health sector, in contributing to community health.

Communities are the lowest level of governance in Nigeria, with well-organised traditional and other formal structures for the provision of multisectoral services, including justice and health systems, which have evolved through the pre-colonial, colonial, and post-colonial era (18). Many formal and informal health providers and facilities exist at the community level at that *“interface between community realities and health system elements, where health services, health workers, community dynamics and actors, and cultural norms and practices interact and promote improved health outcomes*” (19, 20). The tapping of their full potential can immensely contribute to an overall strengthening of the health system, especially helping to significantly improve the provision, access, and use of appropriate health services at the lowest grassroots level, which communities represent (21). Hence, it is important to understand how the health system (HS) is integrated in the communities and how other sectors are harnessed for mainstreaming health for holistic health provision and use.

Nigeria has multisectoral aspirations for health and captures this in its Health in All Policies (HiAP) mandates (22). It recognises that this is a key pathway to achieving the sustainable development goals (SDGs) and UHC and specifically recognises “active community participation and ownership in health planning, implementation, monitoring and evaluation” as a national health policy goal (22). However, it is not clear how much of these aspirations are imbibed by the non-health sector policies or have been translated into actions at the community level.

As part of a series of papers looking at the definition, scope, and realities of communities’ health systems across a range of African settings, our study looks at what multisectoral activities for health are undertaken at the community level, in Nigeria, and what can be done to strengthen them towards achieving universal health coverage (UHC) and in line with the HiAP call. This is also in recognition of the effect of, and the need to respond to the social determinants of health (SDH), using the Health-in-all-Policies approach at the community level as a foundation for overall health system enhancement for achieving SDG3, especially the UHC target.

## Methods

### Study area and setting

Nigeria is a West African country with over 200 million population. The National Population and Housing Census of 2006 placed the population at 140,431,790 (23), but this has since been estimated to have reached 186 million in 2016 (24), and a 2020 estimate of 206 million (25). It runs a federal system of government and is divided into six (non-administrative) geo-political zones, 36 federating states (with a 37th Federal Capital Territory—FCT) and 774 local government areas (LGAs). Some LGAs are urban, and some are rural. LGAs are further divided into varying numbers of wards and communities/villages.

Nigeria is extremely culturally diversified. Half of the population are multidimensionally poor, the majority of whom live in the rural areas (26). Diversity in cultural norms, beliefs, and practises also abound. These impact directly and indirectly on the health sector and the health of the communities (27).

Health and other sectors are governed by Ministries. There are approximately 24–28 Federal Ministries in Nigeria as some get merged by different governments. For example, under the present government, we currently have the Federal Ministry of Health & Social Welfare (previously just the Federal Ministry of Health), coordinated by one minister. These ministries are replicated subnationally as state ministries. In addition, states also have ministries of local government and chieftaincy matters.

Healthcare responsibilities are concurrently devolved across the three tiers of government (national, state, and LGAs). The LGAs have been responsible for all primary healthcare (PHC) and health protection activities, although a recent re-centralisation reform returned stewardship to the state level (28). Other non-health responsibilities of LGAs include pre-primary, primary education and adult education; town planning; water and sanitation, refuse collection and disposal, cemeteries, slaughterhouses, environmental protection, consumer protection, parks and open spaces, sports and leisure facilities, and religious facilities (25).

#### Study design and study area

This was designed as a cross-sectional qualitative study to gather information through review and data extraction from relevant policy documents and interviews from three contextually different states. These states were purposively selected for contextual variations, and second, our research centre has research antecedents in these states, and there were no security challenges at the time of study. Table 1 summarises key socio-demographics and proxy indicators for community health of the states that are relevant to our study.

**Table 1:** Key socio-demographic characteristics of the study states.

Socio-demographic characteristics	Akwa Ibom	Anambra	Kano
Population	3,902,051	4,177,828	5,801,584
No. of LGAs	31	21	44
No. of wards	329	327	463
% of health facility delivery	34.7	90.4	19.2
% of women (15–49 years) with no education	2.9	2.3	56.3
% of children with childhood vaccination card	36.3	56.3	21.3
% of households with basic drinking water service	3.6	0.5	6.2
% of households with basic Sanitation service	49.2	63.3	45.1

Source Nigeria demographic and health survey 2018 (63).

The indicators included are proxies for socio-economic status and cultural beliefs and practises of the study states, and hence pointers to the community health (63).

### Conceptual framework

**Figure 1:**
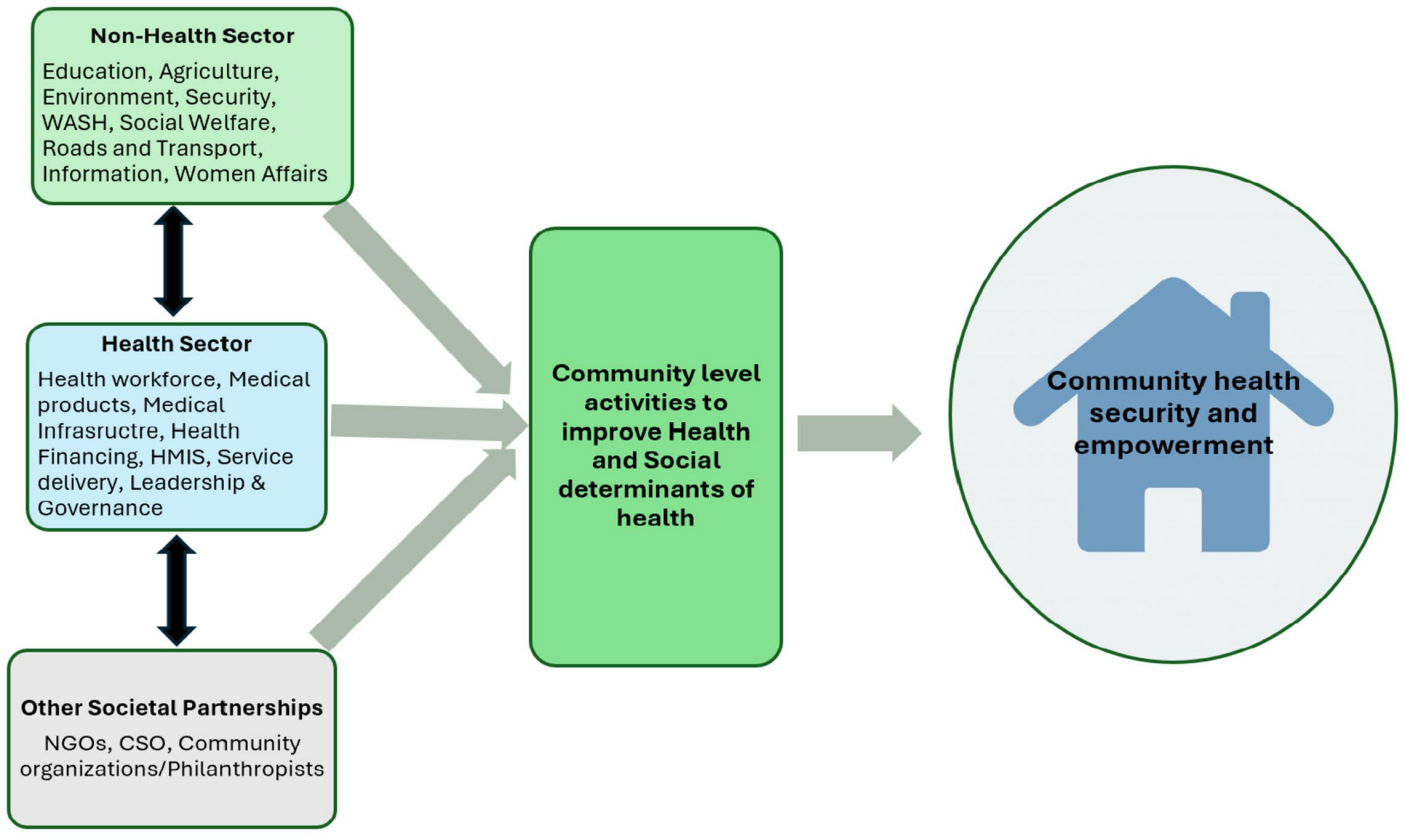
Analytical framework-adapted from the expanded health system building blocks and the WHO PHC operational framework (13 30).

We adapted the expanded health systems building blocks framework (EHSF) for the study (13). The framework includes components which address other modalities peculiar to communities, such as household involvement in health production, and social determinants of health through partnerships and interventions with multiple stakeholders (13). We also applied the PHC operational framework that explicitly recognises multisectoral action as one of the three-pronged approaches for achieving UHC (29). The EHSF shows the dynamism between HS components and explicit community health needs and provides for explicit attention to community-level services, actors, and partnerships necessary to strengthen health systems and provide primary healthcare for all. It recognises the inclusion of community action, household provision of health and partnerships with other non-health sectors, and a multiplicity of stakeholders. The EHSF also shows different potential combinations of collaborations between the non-health sector and other societal partnerships (CSOs, NGOs, community groups, and informal health providers) to directly contribute to community health or indirectly through one or more SDHs. In our study, there is an independent focus on collaboration with the non-health sectors but also recognising other societal partnerships to improve healthcare at the community level (Figure 1).

### Study population, sampling, and data collection

One urban and one rural LGA were selected in each state. At the LGAs, communities that have an ongoing or recently concluded community health programme (which may include immunisation, health education, community mobilisation of women to attend health facilities, distribution of drugs) were purposively selected. In the absence of this, two communities (one urban and one rural) were randomly selected from the LGAs, a total of six communities. A multistakeholder approach was used for participant selection and recruitment, to include relevant stakeholders in the different sectors and community groups—sectoral policymakers (director of public health, Education, Agriculture, private sector), chairpersons of Ward development committees; religious and traditional leaders, community groups (men; women and youth groups), formal health providers (public and private) in the community, informal health providers (traditional birth attendants, patent medicine vendors, bonesetters, and herbalists), non-governmental organisations (NGO) and civil society organisations (CSO) involved in community-level activities.

In the first phase, data were collected through 90 in-depth interviews (IDIs) with health sector policymakers, formal and informal health providers, and community leaders, 12 focus group discussions (FGDs) with community members (service users) and a review of non-health sector policy documents. Documents (national and state levels) included policies and sector strategic plans from health and non-health sectors obtained from the websites of the various ministries.

Following these, non-health sector decision-makers (13 IDIs) were interviewed to ascertain whether their sectoral activities, identified in phase 1, which contributed to community health, were backed by formal sectoral policies. For our study, we considered the following sectors (ministries) relevant with respect to potential collaborations with the health sector: Education and Youth Development; Agriculture; Information and Communication; Works/Transport and Housing; Finance and Economic development; Labour and productivity; Environment and Environmental Health; Women Affairs and Social Development; Water Resources and Rural Development; and Youth and Sports Table 2 summarises the number of interviews and categories of respondents.

**Table 2:** Summary of interviews and categories of respondents.

Respondent category	Akwa Ibom	Anambra	Kano	Interview type (IDI/FGD)
Phase 1 (Health sector and community stakeholders)				
Health sector policymakers	3	2	3	IDI
Health programme managers	2	2	1	IDI
Formal healthcare providers	4	3	4	IDI
Informal healthcare providers	13	7	11	IDI
Intermediary health workers	–	–	3	IDI
Private health sector	–	4	–	IDI
CSO/NGO	2	4	3	IDI
Community or Religious leader	7	5	7	IDI
Community groups (women)	2	2	2	FGD
Community groups (men)	2	2	2	FGD
Phase 2 Non-health sector (public/private) stakeholders				
Education	1	1	1	IDI
Environment/environmental health	1	1	–	IDI
Agriculture	–	1	1	IDI
Security	–	1	1	IDI
Nutrition	1	–	–	IDI
WASH	–	–	1	IDI
Social welfare	–	–	1	IDI
Works and transport	1			
Total interviews per state	39	35	41	103/12
Grand total of interviews in the study	115			

### Data analysis

Available non-health sector policy documents were reviewed to extract data regarding health goals or targets. Interview recordings were transcribed by the interviewers. Interviews conducted in native languages were also translated by the resident research assistants (RAs) who conducted the interviews. Transcripts were thematically analysed using an Excel spreadsheet. A codebook was first developed using themes deducted from the Expanded HS and WHO operational frameworks. Each community activity identified was coded in line with the themes (household production of health, health system building blocks, multisectoral collaboration, community organising and participation, informal health workforce, other societal partnerships, observed benefits from activities, and any emerging themes). Twelve researchers were involved in coding and data analysis. Initially, two transcripts were coded individually by all researchers, after which they came together to agree on any inconsistencies. Each transcript was then coded by two researchers along the themes described above, after which a second meeting was held to harmonise findings. Findings from the review of non-health sector policy documents were triangulated under the multisectoral theme code.

### Ethical considerations

Ethical clearance to conduct this study was obtained from the Health Research Ethics Committee (HREC) at the University of Nigeria Teaching Hospital, Ituku-Ozalla, Enugu State (NHREC/05/01/2005B-FWA00002458-IRB00002323). Written, informed consent was obtained on day of interview, and all interviews were recorded. To maintain confidentiality, recordings were only accessible to the researcher and assistant who transcribed interviews. Anonymised transcripts were stored with identifier codes in a passworded computer.

## Results

First, we present a summary of community activities identified, what collaborations and between what sectors were found. We then outline what formal backing or not exist to guide these collaborations.

### Community-level health-related activities

Several activities were identified, taking place in communities and at household levels, by multiple stakeholders and sectors (Table 3). Some activities are primarily initiated by the health sector or health sector development partners, whilst some are initiated by non-health sectors (Education, Environment, Agriculture, Security, Women Affairs, Social Welfare, Nutrition, Water, Sanitation, and Hygiene—WASH). Some activities are also initiated by the communities. These non-health sectoral activities contribute to community health, either directly or indirectly, through improving one or several social determinants of health (SDH).

**Table 3:** A summary of health-related activities and sectors identified.

Programmes	Activities undertaken	Sector/department involved
Maternal and child health programmes	Antenatal and postnatal services, immunisation family planning activities	Health Private Women Affairs
Community security	Provision of security in the community, health facilities, and other public areas	Private sector Community
Health insurance programmes	Free enrolment of community members in the health insurance scheme	Health Private Community
Health education	Prevention of malnutrition, malaria, and anaemia in pregnancy programmes, education on adolescent health	Education Health Private sector Women affairs
Informal health worker capacity building	Training of traditional birth attendants (TBAs) and voluntary community mobilisers (VCMs)	Health NGO
Health screening	Breast cancer screening, free eye screening, and treatment	Private sector
Food and drug distribution	Distribution of free drugs and mosquito nets; food	Education, agriculture Private sector, Philanthropists Nutrition sector
Environmental sanitation	Cleaning of drainages, spraying of insecticides and fumigation, and general environmental sanitation	Environment Community
Community infrastructure	Community health and water projects, Construction of health facility	WASH sector Private sector (Community Philanthropists)

Some of these sectoral activities include the following:

Education: health education and sensitisation on health issues, commonly child abuse and sexual abuse. There were also education and awareness programmes on environmental sanitation and general cleanliness of living surroundings. The education sector in one of the states of the study also has a social welfare unit that also focusses on households and in the process of setting up structures for household/family counselling and conflict resolutions.

The social welfare unit is also part of the *Department of Women Affairs*, where they focus on assisting with vulnerable and less privileged, addressing issues of abuse, domestic violence, and girl child education, in collaboration with the Ministries of Health, Education, and some NGOs.

*Dept. of Works* recently built health centres in some LGAs, and this has improved physical access to healthcare. In addition to the health centres, new residential housing units have also been developed, with clean water made available for the communities.

The *Nutrition department* embarks on health promotion and distribution of therapeutic food and nutrients to malnourished children and pregnant women in communities. Caregivers of these children are also educated on proper nutrition and hygiene practises.

The *Environmental Health Department* is a standalone department in one of the study states but under the Department of Environment in other states. They carry out regular health inspections and educate on waste disposal, tree planting, and water sanitation practises.

*The Agriculture Department* post-COVID embarked on a food security programme for the poor in rural and urban areas. Farmers are assisted with funds and land to start food production. They carry out public education on safe food processing and storage practises.

*The WASH department* is responsible for the provision of portable water, sanitising and ensuring a hygienic environment within the local government area. WASH activities focus on improved access to safe water, particularly in schools and hospitals, sanitation, and hygiene, which include solarisation of boreholes, rehabilitation of hand pumps, construction of latrines, and hygiene promotion. This department is responsible for ensuring that members of the communities have clean water and that health facilities have appropriate waste disposal systems.

*The Security sector* (public and private) ensures law and order in the communities through community policing.

### Types of collaborations identified

Most collaborations were found to be borne out of community needs. There was a mix of the health sector, NGO, non-health sector-initiated health activities, and a few community-initiated activities. However, community members and groups were actively involved in various stages of activities. The majority of activities were government or donor-funded, and a few by private organisations, philanthropists, or the communities themselves.

We present examples of activities which involved collaboration or multistakeholder engagement into four groups: (i) health sector and formal non-health sector; (ii) health sector and other partnerships (private sectors/CSOs/NGOs, informal providers and community groups), (iii) non-health sector and other partnerships, and (iv) community organising. Whilst the first three categories also involved community members and groups, the fourth, community organising, consists of activities which were primarily initiated by the communities and then involved other stakeholders. Some of these collaborative engagements across study states are highlighted in text boxes (1–4) to illustrate the key forms of collaborations identified.

Box 1 **The Health sector and Education sectors in community disease control.**

In Anambra state, the Ministry of Health and its long-standing NGO partner, the Carter Centre, collaborated with the Ministry of Education in community drug distribution for the treatment of lymphatic filariasis and river blindness for 8 years. The programme just concluded in April 2023.

The health sector engaged area education officers (AEOs) from the education sector in nine LGAs with high prevalence, and along with health officers, went into communities and schools in the seven LGAs to carry out testing and drug administration. They also carried out the opportunistic distribution of mosquito nets and health education on keeping their environment clean and avoiding the mosquito-infested rivers. Other stakeholders outside the health and education sectors were traditional leaders, community women leaders, and town union executives.

The programme was financed by the Carter Centre. In addition to contributing to health sector service delivery, financing, and medical products, it also educated and created awareness in these communities; *“the people there were telling us that they were happy that this program was initiated and that we remembered them…..that they thought that these diseases were due to witchcraft…Some of them said that they thought that its maybe one of their brothers or Uncle that sent the illness that they did not know it was the mosquitoes.” (AN2_NHS_02, Education sector).*

As a key respondent reflects, *“The project really helped many people because…in those prevalent areas where we have the mosquito that causes these diseases, the rate reduced drastically…the drug was able to eradicate those diseases or infections because one man that I observed...we noticed that the leg that was affected by Elephantiasis was drying up...” (AN2_NHS_02, Education sector).*

Box 2 **Formal health workers collaborating with informal providers (TBAs) and other community stakeholders for maternal health service delivery and household production of health.**

In Kano state, there were two initiatives by the government and UNICEF. The first was training and integrating selected TBAs to work to offer ANC in formal settings and health facilities, whilst also working in the communities, *“based on qualification, our leader selectively brings local midwives from the society, and then the selected ones underwent training on how to conduct deliveries”(25_KN_UB_IDI_IP).*

The initiative has been sustained through regular periodic training and supervision of the TBAs. They also offer their services in the community, whilst encouraging women to attend formal health facilities. This has led to increased facility delivery and reduced maternal death. As another TBA reflects, *“the training is encouraging us, and, before most of the women, when they give birth at home they do not care to go to the hospital but now as a result of our work they do come to the hospital,... and now even giving birth at home is very rare...” (18_KN_RU_FGD_CGW-R6).*

The second initiative was the recruitment of selected (by the community) community members to offer health education to households and also mobilise pregnant women to attend facility ANC and delivery. These were known as volunteer community mobilisers (VCMs), although they have to meet certain criteria and are paid a stipend. They interface between the formal, informal providers and community members. They encourage native midwives to refer pregnant women to the facility, visit the health facilities to report any health concerns in the community and engage with the village heads to discuss health-related problems in the communities, and have come to be respected in the community; *“we act like advertisers in the community hoping that whatever intervention/development we bring, the people accept wholeheartedly...we first observe the surrounding of an individual Household, hygiene and carefully make remarks politely where necessary, we inform them about the importance of keeping their bodies and surrounding clean.... The next thing we observe is whether there is a pregnant woman in the house, if indeed there is, we encourage her to be attending antenatal care, we go to the extent of accompanying her to and from the hospital to motivate her”* (*29_KN_UB_IDI_IHW*).

In Akwa Ibom state, a similar government and NGO led initiative to train some TBAs and ‘community informants’ to work with the formal health facilities; *“I go to community informant training as well as TBAs training at the health center, that is being organized by the government where expert doctors are brought in to lecture on pregnancy and childbirth and how to go about ensuring the safety of mother and child, including the knowledge about tools to use during this process” 06_AK_UB_IDI_IP (TBA).*

Box 3 **Social Welfare unit of the Education sector partnering with other non-health sectors for health-related activities.**

In Akwa Ibom State, the Social Welfare unit is situated in the Department of Education and are involved in various partnerships with different non-health sectors and NGO, but with a focus on the health of the community.

Depending on what community activity, they partner with departments of information; youths, sports, and culture; environment; agriculture and women affairs (the gender-based violence unit). They also partner with the Clinical Care and Clinical Research Nigeria (CCCRN). Their activities include the following:

- Community sensitisation on child and female education and prevention of child abuse and domestic violence.
- Identifying abused and malnourished children in the community and referring them to health facilities.
- Sensitising the community on environmental and household hygiene.
- Specifically partner with CCCRN to support families with children living with HIV/AIDS.
- Opportunistic sensitisation and education of youths and young adults during activities of the Ministry of sports.
- Health education, to summarise this; *“our most recent campaign was the world menstrual day which held on the 28th of May 2023. We Organized ourselves along with the wife of the chairman of the council and visited secondary schools where we taught them about proper menstrual hygiene and gave out sanitary pads and some reading materials on the subject. Our social welfare unit places a lot of focus on regular sensitization of young adults to child abuse, sexual abuse, teenage pregnancies and other vices that may affect them. The social welfare unit is also in the process of creating a family court in the local government that will allow quick resolution of family conflicts and give us the platform we need to counsel families” (AK2_NHS_01-Social welfare/Education sector).*

Given the wide network of partnerships, accountability is ensured through joint monitoring by the National Mass Education Commission, Ministry of Education, and Ministry of Women Affairs.

Box 4 **Community-organised security outfit to maintain law and order, which has contributed to improving service delivery and SDHs.**

In Anambra state, seven communities which make up one of the LGAs came together and formed a security organisation 5 years ago.

Through the youth chairman, each community nominated members who would form part of the organisation. A committee was also set up to oversee the activities of the organisation. They engage in community policing, employing their inherent knowledge of their communities. They hold meetings biweekly with the formal (government) police to discuss cases they are not able to handle and for accountability.

Community groups and members provide them with information to work with but do not have any other collaborations with other sectors.

Through maintaining law and order, service delivery has improved, “*in my area, there is a hospital where armed robbers always disturb them there, but since we started working, all these have reduced drastically and the staff can now go to work without fear and patients can easily visit the hospital for treatment any time …..Also, when they go for programs to share medications and other medical products, we also try to ensure their protection so that these things do not get snatched or people interrupt or disturb them” (AN2_NHS_01).*

Other non-health sector activities provided by this group, which, however, address SDHs, include the following:

- Enforcing the Anambra state sanitation law of general monthly cleaning of the environment, at the community level, which has improved adherence and hence cleaner environments.
- Policing of community refuse disposal—they arrest community members who inappropriately dispose of refuse in gutters, for instancing, which blocks the drainages and encourages breeding of mosquitoes.
- When informed, they visit markets to ensure that food sellers are engaged in proper hygiene practises and also prevent the sale of adulterated food and drinks.
- Policing the taking of illicit drugs by community youths.

#### Formal health sector and formal non-health sector collaboration

This programme was successful in interrupting the transmission of river blindness in Anambra state, and other intervention states (30, 31). The Health sector and Education sectors in community disease control shown in Box 1.

#### Health sector and other societal partnerships (private sector, NGOs, CSOs, and community groups)

Our findings show that this combination of collaboration is usually initiated by the government (i.e., formal health sector) and NGOs. Formal health workers collaborating with informal providers (TBAs) and other community stakeholders for maternal health service delivery and household production of health shown in Box 2.

#### Formal non-health sectors and other partnerships

These include activities of non-health sectors independently or with another non-health sector or other partnerships, which inadvertently contributed or improved community health or SDHs. Social Welfare unit of the Education sector partnering with other non-health sectors for health-related activities shown in Box 3.

#### Formal health sector and community organisations

Community organised security outfit to maintain law and order which has contributed to improving service delivery and SDHs shown in Box 4.

### Were collaborations backed by formal sector policies?

Our findings from a review of non-health sector policy documents show that whilst some sectoral activities are backed by their sectoral policies, however, collaborative engagements are not explicitly spelt out. There is a paucity of clear pathways or guidelines in the non-health sector policies identified.

The current national policy on education does not spell out any collaborative actions with the health sector (32). The Women Affairs department is guided by the National Gender Policy (2008), of which the only stated intended collaboration with the health sector is to work with the FMOH to develop guidelines on gender-based violence (33) and a state-level respondent corroborates some level of implementation, *“Oh, yes, our activities are backed up by policies from the ministry of social welfare and state departments that focus on violence against persons and gender-based violence.” (AK2_NHS-01, Subnational female policy maker).* The national environmental policy (2016) intends to “…encourage and promote the use of appropriate technology and local expertise to raise community awareness, standards of health and safety education …” (P.0.42) as one of its cross-sectoral objectives but did not outline any explicit actions nor activities through which this will be achieved in collaboration with the health sector (34). *A sector policymaker reflected that, “…the activities are backed by state policies and well as state and national laws. Example, Environmental health and practice regulations, the law provides that there is one environmental health worker to 10,000 Nigerian citizens” (AK2_NHS-04, sector policymaker).* A different view from the environment sector of another state, *“No definite policy, but the governor called for proposals and people submitted, so he approved the one that supports his vision.” (AN2_NHH-03, sector policymaker).*

The National Food and Nutrition Policy (2001) guides the Nutrition sector incorporates multisector actors, including health, in its policy development, but there are no explicit pathways for collaboration in their healthy lifestyle policy objectives (35). The national policy on Agriculture (2001) is also silent on any health-related collaborative objectives (36).

At the subnational level, the Kano State Water and Sanitation was the one available state policy with a clear mandate of collaboration with the State Ministry of Health to undertake water surveillance and monitoring, under the Kano State Water and Sanitation Sector Reform Law (2019) (37). The Kano State water surveillance initiative is a good example of a subnational initiative that could be scaled up across other states. However, the federal governance structure in Nigeria does not adequately provide for horizontal adaptation of policies across states because of the executive decision-making power that resides with states. In addition, states can adopt, adapt, reshape, or reject out rightly national-level policies. Hence, transferring principles and actions that have worked from one state to another is purely at the behest of the state governors who retain executive decision-making in their states. However, where a policy or programme content involves result-based incentives, states have been known to align. An example is the Saving One Million Lives (SOML) maternal and child programme, where state implementation results were posted on national league tables and disseminated at stakeholders’ meetings, and high-performing states were rewarded with financial incentives. This influenced the least-performing states to improve their implementation objectives (38, 39).

Despite evidence to show that resources to optimise the control of a number of health conditions lie beyond the health sector, it continues to be seen as the problem of the health sector alone. The HiAP agenda has aimed at re-framing health, as a responsibility of multiple sectors, but there remains inadequate uptake of this notion by non-health sectors. Hence, most policies with multisectoral aspirations remain domiciled in the health sector (40). Before the multisectoral action plan for NCDs, there have not been any explicit formal policies for collaboration. There are independent sectoral programmes that indirectly influence health by addressing one or other social determinant of health, but there are no explicit mandates for a clear cross-sectoral collaboration. This may be due to several policy implications, such as which sector will domicile or host the policy, budgetary, implementation, monitoring, and evaluation responsibilities, which then raises the second reason, which is the resources (financial, workforce, etc.) to drive multisectoral action.

*Methods of collaboration* usually include initial sensitisation meetings between potential collaborators, following which sectors and stakeholders who wish to collaborate indicate their interest.

### Facilitators to collaborations and partnerships

#### Community structures

The community structures that aid partnerships rest on the ward development committees, village heads, and religious heads who make themselves available by working with government and multinational agencies for health. Across the board, support from recipient communities was key, even where clear needs were identified, “…*the structures in the community that help our work are the village council and traditional rulers. They pave the way for us to enter the community and help introduce us to other groups in the community like the women groups, the youth groups and young adult groups….” (AK2_NHS-01).* In addition, the participation of community members and groups contributes to facilitating the activities facilitate. Where activities are recurrent, communities have been useful in sustaining such activities.

### Leadership and governance of community-level multisectoral activities

Wards or villages that make up each community are represented in the leadership structure of most communities, by having a member in the executive committee, *“You know there is no way people from the same ward or village will occupy all the leadership positions in a community. It must be somehow distributed that each village or ward will have their own representative in the project” (14_AN_UB_FGD_CGW-R1).* In some communities, in addition to this community health leadership structure, traditional and religious heads (ward head, district head, Imams, and community elders) are also automatically included in the leadership structure. Where health activities are government-funded, leadership and governance structures mainly reside in the formal sector, *“The hospital management board is in charge of the medical help, and there is a record book that they take the information of the people that benefit from the help, for verification (03_KN_RU_IDI_IP).”* Some projects are particular about prioritising community voices, *“All the community groups are meant to be involved in the community group discussion, the men, the women, the youths, the vulnerable are made to discuss what they need. After that, we embark on voting and after that the need is chosen….. We prioritize the voices of the vulnerable among them, like the older adults, women, physically challenged, etc.” 11_AK_UB_IDI_PM_CSDP.*

In addition to this organic community leadership structure, there are also informal provider associations (PMV Association; TBA association) with clearly outlined leadership structures, membership registration, and disciplinary guidelines.

### Constraints

#### Funding

Availability of funding was frequently identified by respondents as a key factor that drives cross-sectoral collaboration and constraints on activities when funding is inadequate.

## Discussion

We discuss the key findings from this study, which include the multiplicity of health and related activities at the community level and the actor and community commitment and involvement in these activities. We also discuss the multistakeholder approach to these activities and the inadequate formal multisectoral backing for improving and validating these activities.

### The multiplicity of activities at the community level

Community-level health-related activities are usually in response to community needs, which may have arisen organically or identified through different forms of needs assessment by different bodies, as seen in our findings. The nature of the origins of communities in Nigeria and other countries of the African region and the way they have evolved over several decades form a background for how these activities originate and are structured and organised (3, 18, 41). In Ethiopia, several community-level activities (community-based nutrition, family planning, and maternal and child health services) within and outside its popular health extension programme (42). In Ethiopia, a comprehensive package of care programme, including disease prevention, environmental sanitation and hygiene, family planning (FP), and nutrition, targeting households, women, and children (a total of 16 components) was implemented at the community level and integrated through full-time extension workers, with support from other actors.

### Multistakeholder involvement in activities

The majority of activities, irrespective of original initiators, involved diverse actors (individuals and groups) from various sectors and community groups, including state and national levels. Informal cross-sector collaborations have been highlighted in a preceding paper (21).

Diversity of actors is a key feature of community-level health activities and the community subsystem (14). An integrated family planning and nutrition programme in Ethiopia brought together an array of community groups and governmental stakeholders and specifically recruited and trained youth groups to target and mobilise adolescents and out-of-school for family planning which contributed to the success of the programme (42). However, a community initiative in Kenya, which directly targeted youths, was not as successful and recommended models for improved youth engagement and participation (43). A programme in a Zimbabwean district, to strengthen the CHS for better delivery of HIV treatment, support, and care, also included the police, farmers associations, and women’s action groups (44).

The use of local governments and community structures in stimulating health service patronage, monitoring health service delivery, advocating for and mobilising resources for health infrastructure and healthcare management, has been a recommendation for multisectoral attention (Options, 2018). This has been made manifest in several programmes, such as Northern Traditional Leaders Committee for Polio and Primary Health Care (NTLC) (VaccinesWork, 2021), Community-based Health Planning and Services (CHPS) in Ghana (African Union Commission, 2021), and the Progressive Primary Health Care Network (PPHCN) (a network of local community health organisations) supported by the National Medical and Dental Association, poised to strengthen project cross-fertilisation and collaboration for primary healthcare in South Africa (45).

Communities are usually represented by the ward development committees (WDCs) and health facility committees (FHCs) in health-related matters. In addition, some community-based organisations and faith-based organisations represent various community groups in decision-making activities. They, to various extent, ensure that community voices are heard and sometimes used in decision-making. However, the level of community involvement is still suboptimal at the federal and state levels but is amplified at the local government and ward levels. Hence, ways and means of ensuring greater community involvement in decision-making at all levels of government remain a matter of national discourse. In the national multisectoral plan, communities are represented by the local government steering committee (40).

Observed partnerships were also *ad hoc*. The sustainability of these partnerships would require strong leadership and governance structures. Partnerships must include a planned transition of responsibilities to the communities to inspire ownership amongst the people (46). This was evident in the Global Polio Eradication Initiative Nigeria, which involved religious leaders playing significant roles in facilitating health promotion, leading to the defeat of polio in the country (18). The willingness of community members to own health projects is important, but this can be achieved if those who design projects take into consideration the insights and belongingness of the communities (47, 48). Towards strengthening and harmonising leadership and governance structures, a distributed leadership model is proposed, which promotes the co-creation, by stakeholders, of a shared understanding of their daily interactions for healthcare activities (49). The joint monitoring by the National Mass Education Commission, Ministry of Education, and Ministry of Women Affairs in Akwa Ibom State (Box 3) is a good example of a strategy that could be strengthened with a framework or formal guideline for sustainability. Leadership and governance of diverse stakeholders require some level of formalisation and distributed leadership to achieve aspired goals.

### Inadequate or absence of formal non-health sectoral policies

The observed diverse multistakeholder involvement, however, was not explicitly and adequately backed by formal national or subnational multisectoral policies and guidelines. Hence, most activities were *ad hoc* and not sustained. Formal sector-wide collaboration presently depends mainly on the willingness of the local decision-makers and the availability of funding for such activities. There are currently frameworks for ensuring that such partnerships are sustained beyond specific projects and initiatives. They include a multisectoral plan that has been developed by the FMOH in 2019. Although this plan focusses on non-communicable diseases, it is a comprehensive plan that, if optimally implemented, can be transferred to other health conditions and activities. The plan was modelled on the WHO Global NCD Multisectoral Action Plan (50). It adopts a Health in All Policies approach, emphasising collaboration across health and non-health sectors, and aligns with the Sustainable Development Goals (SDGs) and the National Strategic Health Development Plan II (NHSDP II). The plan focusses on cost-effective interventions identified as “best buys” by the WHO. It recognises existing and ongoing efforts by non-health sector MDAs that address some of the risk factors for priority NCDs and notes the lack of a strategic plan of action to address these risk factors through a multisectoral approach. The plan then apportions specific policy actions and activities to public and private non-health sectors, including roles in advocacy, community engagement, resource mobilisation, development/design and implementation of health promotion programmes/interventions, and enforcement of legislations for control of NCDs. In addition, the Ward Development Committees as encapsulated in the Strategy for strengthening PHCs in Nigeria, the guidelines for implementing the Basic Healthcare Provision Fund, the National Health Policy, and the Renewed Hope Health Agenda of the federal government all provide frameworks for sustaining such partnerships. The applicability of the multisectoral approach in the health sector is found in literature. In Spain, 48 SDG targets were identified as relevant to urban health, and the plans to achieve them were made by synergising the comparative advantages of diverse sectors (51). The sectors that were brought together included urban and transport planning, environment, legislature, nutrition, education, and health, amongst others. The study concluded that health and wellbeing within urban areas improved rapidly when multiple government agencies came together to achieve good health for the urban population, as compared to slowly paced improvement when they acted in silos. In Philippines, the application of a multisectoral approach to health informed the establishment of three important elements that can guarantee the successful application of multisectoral actions and policies, which include clear outcomes, resources, and designated roles (52). In very recent times, COVID-19 made clear the importance of a multisectoral approach to health systems, as was seen in concerted sectoral efforts to contain the pandemic (53, 54). Commonly experienced across most reported multisectoral approaches to health is the usual national-level concentration, with less attention paid to communities.

The primary health structure is community focussed, and Nigeria’s primary healthcare, after evaluation by the Primary Healthcare Performance Initiative, is judged to be of low quality and amongst the worst globally (55). This leaves many with the question about quality community health services, especially with a suboptimal primary healthcare system in place. However, health remains a right, inclusive of those living in the grassroots. As already mentioned, reversing these concerns can be made possible when different stakeholders at horizontal and vertical levels come together to make community health a core interest.

The success of the community-level programmes in Ethiopia was ascribed to existing structures—strong community ownership, strong intersectoral collaboration (ISC) with federal district structures/development partners, and a strong HMIS (42). This project also faced constraints with funding and optimal stakeholder coordination. However, the lessons learnt as outlined above may provide some transferable principles to Nigeria, where there are also resource constraints.

Similarly, in Rwanda, an MSC nutrition programme using various interventions was backed by an existing policy aspiration. The project used a cluster community-organising model that worked through community networks. Through this model, it helped mobilise a vast array of local groups—formal and informal, for-profit and not-for-profit, governmental and non-governmental, to lead programming from initial needs assessments to programme planning to collaborative implementation. This promoted joint cooperative action of like-minded groups to address local needs, increased coverage and ensured participation of grassroots groups that otherwise would generally not be involved in community health programming (56). Such a model could also be adapted to the Nigerian context and piloted.

Our study is limited by not capturing all the sectors with roles to play in health, such as power, finance, and budget. The ministries of finance and budgeting at both national and subnational levels are responsible for fund allocation to other sectors, based on the budget presented. Although these sectors may not necessarily directly address a social determinant of health, their commitment and buy-in may favour resource allocation to health and non-health sectors towards improving community health.

Although some of these sectors are not found at the level of the communities, their inclusion might have added some insights into our findings. Hence, we encourage subsequent studies to expand the studied sectors. In addition, we noticed poor documentation practises across the sectors, which constrained the review part of this research.

## Conclusion and recommendations

A clear commitment to formal multisectoral collaboration for health at the community level is required as part of re-engineering primary healthcare towards UHC and achieving SDG3. This needs to be done through explicitly intentional policy reforms, with adequate community representation during policymaking and their implementation, through identifying, promoting, and co-financing actions that require collaboration between two or more sectors, that will enhance joint capacity and benefits (6, 57). Nigeria currently operates a “basket funding” to pool financial resources at the national level for healthcare purchasing. It pools resources from donors, the private sectors, and other stakeholders (58). This model could also be piloted and evaluated at the community level. The Nigeria National Multisectoral Action Plan (NMSAP) for NCD control sets out a framework to monitor specific health outcomes of priority NCDs and risk factors, leadership, and governance and also sets out a framework for multisectoral coordination (40). This can be adapted across other multisectoral health programmes for health. Adequate monitoring and evaluation of multisectoral action is also a necessity for sustainability. Where multisectoral collaboration intent is in place and implemented, this could be evaluated by measuring specific health outcomes, social determinants of health (59), intermediate objectives such as access and service delivery (60, 61) or ultimate health system goals, e.g., equity. However, a systematic review of multisectoral interventions has shown that many studies proposed mechanisms explaining how multisectoral interventions for health could lead to the intended outcomes, but none used realistic evaluations to assess these (62).

## Data Availability

The raw data supporting the conclusions of this article will be made available by the authors, without undue reservation.
